# Protective effects of α-ketoglutaric acid on palmitic acid-induced deterioration in sheep endometrial epithelial cells via ferroptosis inhibition

**DOI:** 10.3389/fvets.2025.1617348

**Published:** 2025-11-12

**Authors:** Ziyi Bai, Qinyuan Fang, Shubin Li, Mengxuan Jia, Rongrong Zhang, Chunyu Wang, Zhenli Wu, Gang Liu, Yongbin Liu

**Affiliations:** 1 College of Life Science, Inner Mongolia University, Hohhot, China; 2 Department of Geriatric Medical Center, Inner Mongolia People’s Hospital, Hohhot, China; 3 Clinical Medicine Research Center, Affiliated Hospital of Inner Mongolia Medical University, Hohhot, China; 4 Department of Chemical Engineering, Inner Mongolia University of Technology, Hohhot, China; 5 College of Animal Science, Inner Mongolia Agricultural University, Hohhot, China

**Keywords:** sheep endometrial epithelial cells, α-ketoglutaric acid, palmitic acid, histone trimethylation, ferroptosis

## Abstract

**Introduction:**

Emerging evidence indicates that dysregulated palmitic acid (PA) homeostasis plays a key role in inducing lipotoxicity and cellular dysfunction in mammalian endometrial epithelial cells. While this phenomenon has been documented in bovine models, the underlying mechanisms of PA-induced toxicity in sheep endometrial epithelial cells (SEECs) remain poorly understood. Moreover, effective strategies to counteract PA-mediated damage in SEECs have yet to be fully explored.

**Methods:**

In this study, we investigated the protective effects of α-ketoglutaric acid (α-KG), a central metabolic intermediate in the tricarboxylic acid (TCA) cycle, against PA-induced cellular impairment in SEECs. Functional assays were performed to assess changes in cell viability, proliferation, migration, lipid accumulation, cell cycle progression, DNA damage, histone trimethylation, and apoptosis. Integrated transcriptomic and metabolomic analyses were conducted to elucidate the molecular pathways involved.

**Results and discussion:**

Our results demonstrated that α-KG markedly alleviated PA-induced cytotoxicity. Specifically, α-KG enhanced cell viability, restored proliferative and migratory capacities, promoted cell cycle progression, and attenuated lipid accumulation, DNA damage, histone trimethylation alteration, and apoptosis. Multi-omics profiling, supported by ferroptosis-specific assays, revealed that these cytoprotective effects were predominantly mediated through the suppression of PA-induced ferroptosis. Collectively, our findings provide novel mechanistic insight into the role of α-KG in mitigating lipid-induced cellular stress and establish its therapeutic potential as a metabolic modulator. This study not only advances our understanding of ferroptosis in reproductive cell biology but also opens new avenues for targeted interventions against lipotoxic damage in endometrial tissues.

## 1 Introduction

With rapid socio-economic development, urbanization, and growing awareness of health-conscious consumption, consumers are increasingly seeking high-quality meat from small ruminants, such as sheep and goats (1, 2). The rising demand for premium dairy products with superior organoleptic qualities, enhanced health benefits, and optimal nutritional properties has significantly driven advancements in sheep farming, breeding, and production systems (3).

It is well known that diet has been recognized as a key determinant for the efficiency of feed utilization, growth performance, carcass attributes, and sensory quality of sheep meat (1, 3, 4). Current sheep feeding systems consist of grassland grazing, indoor feeding, and pasture grazing supplemented with dietary additives (3). While pasture-based feeding systems are cost-effective and environmentally sustainable, intensive feed-based systems accelerate fattening and allow for the regulation of meat fat content. Consequently, traditional grazing practices are being replaced by indoor feeding systems (5). However, stall-feeding, characterized by limited mobility and dietary modifications, impacts sheep health by reducing oxidative stress resistance and compromising immune function, thus posing challenges to the industry sustainability (5). During the fattening process, high-concentrate diets are provided to meet nutritional requirements and sustain productivity. Compared to pasture-raised lambs, those finished indoors or under stall-feeding systems tend to exhibit higher average daily gain and carcass yield. However, these strategies also lead to a more intense flavor profile, increased intramuscular fat content, and undesirable saturated fatty acid (SFA) compositions (1, 2, 6).

The health risks associated with excessive SFA intake often overshadow the nutritional benefits of ruminant-derived fats. This concern primarily arises from the presence of thrombogenic and atherogenic fatty acids, which have been implicated in an increased risk of cardiovascular disease, obesity, and hypercholesterolemia (7). Additionally, excessive SFA intake may contribute to carcinogenesis and mutagenesis through the formation of harmful byproducts during processing (7).

Palmitic acid (PA), a major SFA found in animals, plants, and microorganisms, constitutes approximately 20–30% of the total FA in human and animal tissues. Its metabolism is tightly regulated through endogenous synthesis via *de novo* lipogenesis (DNL) and dietary intake (8, 9). Under normal physiological conditions, dietary PA is rapidly metabolized, incorporated into cellular membranes, or utilized for energy production, making it an essential nutrient. PA also plays a crucial role in post-translational modifications (PTMs), such as palmitoylation, DNA methylation, histone methylation, and acetylation (8, 10–13). At moderate levels, PA promotes *β*-oxidation and ATP production, thus supporting mitochondrial function and cellular homeostasis. On the other hand, excessive PA accumulation triggers lipotoxicity, leading to disrupted calcium homeostasis, increased reactive oxygen species (ROS) production, mitochondrial membrane potential (MMP) collapse, impaired oxidative metabolism, and mitochondrial DNA damage (14). These adverse effects exacerbate endoplasmic reticulum (ER) stress, oxidative stress, autophagy, apoptosis, and ferroptosis (14).

Dysregulated PA homeostasis has been implicated in various pathological conditions, including atherosclerosis, diabetes, obesity, carcinogenesis, inflammation, insulin resistance, metabolic syndrome (MetS), and neurodegenerative diseases (15). Furthermore, disrupted PA metabolism has been shown to negatively impact the development of mammalian oocytes and granulosa cells, ultimately leading to reduced fertility (16, 17). In 2018, Chankeaw et al. (18) reported for the first time that PA exposure compromises the viability and proliferation of bovine endometrial cells while promoting lipid accumulation, pro-inflammatory cytokine release, and apoptosis. Similarly, Li et al. (19) found that excessive PA exposure induces inflammatory responses in bovine endometrial cells by activating the oxidative stress-mediated NF-κB signaling pathway. PA overload has also been shown to promote lipid accumulation and increase histone 3 lysine 9 trimethylation (H3K9me3) in bovine endometrial epithelial cells (20). Additionally, PA-induced mitochondrial dysfunction, characterized by oxidative stress and autophagy, has been observed in these bovine endometrial cells (21), supporting the effect of PA exposure on inducing the deterioration of mammalian endometrial epithelial cells. However, the precise mechanisms underlying PA-induced dysfunction in sheep endometrial epithelial cells (SEECs) and potential strategies to mitigate PA-induced lipotoxicity in SEECs remain poorly understood.

As a crucial intermediary metabolite in the tricarboxylic acid (TCA) cycle and a product of glutaminolysis, α-ketoglutaric acid (α-KG), also known as 2-ketoglutaric acid or 2-oxoglutaric acid, is biosynthesized through the conversion of glucose or oxaloacetate in combination with pyruvate (22). As a precursor for glutamine synthesis, α-KG serves as a rate-determining metabolite in the TCA cycle, playing a pivotal role in regulating oxidative phosphorylation (OXPHOS) and mitochondrial metabolism across various organisms (22). Beyond its metabolic roles, α-KG orchestrates numerous biological processes, including gene transcription, protein translation, antioxidation, autophagy, and immune cell differentiation (22–24). In addition to its metabolic functions, α-KG exhibits tumor-suppressive properties through multiple mechanisms, including attenuating cellular activity, inhibiting tumor invasion, suppressing angiogenesis, and inducing apoptosis (25–28). Moreover, α-KG serves as a critical epigenetic regulator, influencing histone and DNA methylation by acting as a cofactor for Jumonji C domain-containing histone demethylases (JMJDs) and Ten-eleven translocation (TET) DNA demethylases (23, 29). This dual role in metabolism and epigenetic regulation highlights the complexity of α-KG’s biological functions and its potential as a therapeutic agent in various pathophysiological contexts.

Our previous research demonstrated that α-KG enhances the developmental competence of sheep oocytes by promoting mitochondrial activity and intracellular glutathione (GSH) production while mitigating oxidative stress, MMP collapse, DNA damage, and apoptosis (30). Based on these findings, we hypothesize that α-KG may similarly protect SEECs from PA-induced lipotoxicity. In this study, we investigated the protective effects of α-KG on PA-induced SEECs deterioration. To elucidate the underlying molecular mechanisms, transcriptomic and metabolomic profiling were performed, complemented by Gene Ontology (GO) and Kyoto Encyclopedia of Genes and Genomes (KEGG) pathway enrichment analyses. Through this comprehensive approach, we aimed to elucidate the mechanistic basis of α-KG’s protective role in PA-induced SEECs dysfunction. Our findings not only enhance the understanding of α-KG’s regulatory functions but also provide novel insights into its potential therapeutic applications for mitigating PA-induced metabolic disturbances in SEECs.

## 2 Materials and methods

### 2.1 Ethics statement

All animal procedures conducted in this study were approved by the Ethics Committee of Inner Mongolia University and adhered to institutional and national guidelines for animal research.

### 2.2 Chemicals

Unless otherwise specified, all chemicals, culture media, and supplements were obtained from Sigma-Aldrich (Shanghai, China) and Thermo Fisher Scientific (Beijing, China).

### 2.3 Isolation, culture, and identification of SEECs

A modified two-step enzymatic digestion method, based on previous studies (31), was employed to isolate SEECs. Uteri were collected from 2-3-year-old Mongolian sheep sourced from a local slaughterhouse in Hohhot, Inner Mongolia. The uterine horn was excised, and the uterine caruncle was dissected and placed into ice-cold normal saline containing 500 U/mL penicillin and 500 ng/mL streptomycin (15,070,063, Thermo Fisher Scientific, Beijing, China). The ovaries were transported to the laboratory at 4 °C within 2 h. Upon arrival, the uterine caruncle was washed with 70% ethanol to minimize contamination, followed by several rinses in sterile normal saline. The caruncle tissue was then cut into small pieces, washed with Dulbecco’s phosphate-buffered saline (DPBS, 14190250, Thermo Fisher Scientific, Beijing, China), and incubated with 1 mg/mL type IV collagenase (17,104,019, Thermo Fisher Scientific, Beijing, China) at 37 °C for 40 min. A subsequent incubation with 0.25% trypsin–EDTA solution (25,200,072, Thermo Fisher Scientific, Beijing, China) at 37 °C for 15 min was performed. Digestion was terminated by adding an equal volume of fetal bovine serum (FBS, 10091148, Thermo Fisher Scientific, Shanghai, China), and the suspension was centrifuged at 1500 rpm for 5 min. The resulting pellet was resuspended in Dulbecco’s modified Eagle medium/F-12 (DMEM/F-12, 11,320,033, Thermo Fisher Scientific, Beijing, China), supplemented with 10% FBS, and passed through a 70 μm filter (352,370, FALCON, Beijing, China) to remove the cell clumps. After centrifugation at 1500 rpm for 5 min, SEECs were cultured in the same medium in a CO_2_ incubator (38.5 °C, 5% CO_2_). The medium was refreshed every 3 days. Immunofluorescence (IF) staining for CK18 (Supplementary Figure 1A) and Vimentin (Supplementary Figure 1B) confirmed the purity of SEECs. A list of antibodies used is provided in the Supplementary Table 1.

### 2.4 Drug treatment

To expose SEECs to PA, a working concentration of 200 μM was employed. Considering that PA is highly hydrophobic and poorly soluble in aqueous buffers such as DPBS, a 400 mM PA (P5585, Sigma-Aldrich, Shanghai, China) stock solution was initially prepared in 100% ethanol. In parallel, low endotoxin bovine serum albumin (BSA, A8850, Solarbio, Beijing, China) was dissolved in serum-free DMEM/F-12 medium to a final concentration of 13.5% (v/v), which served as a carrier to stabilize PA and enhance its bioavailability. For conjugation, PA and BSA were combined at a molar ratio of 1:2.5 (PA: BSA), resulting in a 5 mM PA-BSA stock solution. The mixture was subjected to sonication, followed by gentle nutation at room temperature for 3 h, filtered through a 0.22 μm membrane for sterilization, aliquoted, and stored at −80 °C until further application (32).

Cells were then treated with PA alone or in combination with α-KG (328–50-7, the membrane-permeable form of α-KG without non-toxic to mammalian cells, Sigma-Aldrich, Shanghai, China) at concentrations of 1.5, 3, and 6 mM for 24 h. The negative control (NC) group was established by treating SEECs with 200 μM PA together with an equal volume of DPBS in place of α-KG, thereby maintaining identical PA exposure and treatment volumes across all groups. The chosen concentrations of PA and α-KG were based on previous reports, with minor modifications to optimize the experimental conditions (21, 30).

### 2.5 Cell viability assessment

Cell viability was assessed using the MTT and lactate dehydrogenase (LDH) assays (33). For the MTT assay, MTT reagent (C0009S, Beyotime, Shanghai, China) was added to the culture well of SEECs post-drug treatment, and cells were incubated in a CO_2_ incubator (38.5 °C, 5% CO_2_) for 4 h. After the addition of dimethyl sulfoxide (DMSO, 67–68-5, Coolaber, Shanghai, China) to solubilize the Formazan product, absorbance was measured at 570 nm using a microplate reader. For the LDH assay, supernatants of SEECs post-drug treatment were collected, and LDH activity was quantified according to the manufacturer’s instructions (C0017, Beyotime, Shanghai, China), with absorbance measured at 490 nm. These assays were applied to determine the optimal α-KG concentration for subsequent analyses.

### 2.6 Cell proliferation assessment

To assess the effect of α-KG on PA-induced proliferation inhibition of SEECs, the 5-ethynyl-2′-deoxyuridine (EdU) incorporation assay was performed (33). Following the manufacturer’s protocol, SEECs post-drug treatment was incubated with EdU solution (C0071S, Beyotime, Shanghai, China) in a CO_2_ incubator (38.5 °C, 5% CO_2_) for 2 h, followed by fixation in 4% paraformaldehyde (PFA, P1110, Solarbio, Beijing, China) in a CO_2_ incubator (38.5 °C, 5% CO_2_) for 15 min. The cells were then incubated with 4′,6-diamidino-2-phenylindole solution (DAPI, C0065, Solarbio, Beijing, China) in a CO_2_ incubator (38.5 °C, 5% CO_2_) for 15 min for nuclear staining. Representative EdU staining images were acquired via confocal microscopy, and staining intensity was quantitatively analyzed using ImageJ software (34).

### 2.7 Scratch migration assessment

A cell scratch assay was performed to evaluate the impact of α-KG on PA-induced migration defects of SEECs (33). Accordingly, SEECs were seeded in 6-well plates and cultured in a CO_2_ incubator (38.5 °C, 5% CO_2_) for 24 h. Prior to the drug intervention, SEECs were allowed to grow until reaching a high confluence (90–100%) to ensure consistency. A wound was created by scratching the monolayer with a 200 μL pipette tip. Non-adherent cells were removed by washing with DPBS, and cells were cultured with the fresh culture medium containing DPBS, PA, or α-KG for 24 h. Wound closure was monitored by imaging at 0 and 24 h post-scratching. A cell scratch assay was performed to analyze the potential effect of α-KG treatment on the PA-induced migration deterioration of SEECs.

### 2.8 Oil red O staining

Oil Red O staining was performed to assess the effect of α-KG on PA-induced lipid accumulation in SEECs (21). Briefly, SEECs post-drug treatment were fixed with Oil Red O fixative (G1262, Solarbio, Beijing, China) at room temperature for 20 min, followed by washing with 60% isopropyl alcohol for 5 min. The cells were then stained with Oil Red O solution at room temperature for 15 min and further stained with hematoxylin for 5 min. After microscopic observation, the results were captured and analyzed using ImageJ software.

### 2.9 Cell cycle analysis

To analyze the potential effect of α-KG treatment on the PA-induced cell cycle arrest of SEECs, the cell cycle progression of SEECs post-drug treatment was detected using cell cycle analysis (33). Accordingly, SEECs post-drug treatment were harvested, fixed in ice-cold 70% ethanol overnight, and stained with propidium iodide (PI, MA0334, Meilunbio, Dalian, China) following the manufacturer’s protocol. The cell cycle distribution in the G0/G1, S, and G2/M phases was determined using flow cytometry (BD Biosciences, San Jose, CA, USA).

### 2.10 DNA damage and histone trimethylation evaluation

To assess the protective effect of α-KG on PA-induced DNA damage and histone trimethylation alterations in SEECs, IF staining of SEECs was performed (33). Briefly, SEECs post-drug treatment were fixed, permeabilized with 0.5% Triton X-100 solution (T8200, Solarbio, Beijing, China) at 37 °C for 15 min, and incubated with a rabbit anti-γH2A. X antibody, a rabbit anti-H3K4me3 antibody, a rabbit anti-H3K9me3 antibody, a rabbit anti-H3K27me3 antibody, or a rabbit anti-H3K36me3 antibody at 4 °C overnight. After incubation with the secondary antibody at room temperature for 1 h and DAPI counterstaining, the IF staining intensity of each group was analyzed using ImageJ software. A list of antibodies used is provided in the Supplementary Table 1.

### 2.11 Apoptosis assay

The Annexin V-FITC apoptosis assay was used to evaluate the effect of α-KG on PA-triggered apoptosis in SEECs (33). According to the manufacturer’s protocol, SEECs post-drug treatment were harvested and incubated with Annexin V-FITC (CA1020, Solarbio, Beijing, China) at 37 °C for 5 min. The apoptosis rates of each group were analyzed by flow cytometry within 1 h.

### 2.12 Transcriptomic analysis

To confirm the comprehensive regulation mechanism related to the effect of α-KG against PA-induced deterioration of SEECs, transcriptomic analysis of SEECs post-drug treatment was conducted by the Novogene Biotechnology Company (Beijing, China). Total RNA was extracted using TRIzol reagent (Invitrogen, Carlsbad, CA, USA), and RNA quality was assessed using an Agilent 2,100 Bioanalyzer (Agilent Technologies, Santa Clara, CA, USA). mRNA was enriched using Oligo (dT) beads, fragmented, and reverse transcribed into cDNA using the NEBNext® Ultra™ RNA Library Prep Kit for Illumina® (New England Biolabs, Ipswich, MA, USA). The cDNA library was sequenced on an Illumina Novaseq6000 platform. Bioinformatics analyses were performed using HISAT2 for alignment, RSEM for expression quantification, and DESeq2 for differential expression analysis. Differentially expressed genes (DEGs) were identified with a false discovery rate (FDR) < 0.05 and absolute fold change (FC) ≥ 2. Gene ontology (GO) and Kyoto Encyclopedia of Genes and Genomes (KEGG) pathway enrichment analyses were conducted (35).

### 2.13 Metabolomic analysis

Metabolites were extracted from SEECs post-drug treatment and analyzed using quadrupole time-of-flight mass spectrometry coupled with hydrophilic interaction chromatography by the Novogene Biotechnology Company (Beijing, China). Peak intensities were normalized, and metabolite identification was based on mzCloud, mzVault, and MassList databases. Differential metabolites were identified with variable importance in projection (VIP) ≥ 1, |log₂ fold change| ≥ 1, and *p* < 0.05. KEGG pathway enrichment was performed using Fisher’s exact test (33).

### 2.14 ROS production assay

According to the transcriptomic and metabolomic results, the effect of α-KG on PA-induced ferroptosis was assessed by analyzing the ROS production using a dichlorofluorescein diacetate (DCFH-DA) fluorescence probe. According to the departmental instructions (33), SEECs post-drug treatment were incubated with 10 μM DCFH-DA solution (S0033, Beyotime, Shanghai, China) at 37 °C for 30 min, followed by DAPI counterstaining. After microscopic observation, the DCFH-DA staining intensities were quantitatively analyzed using ImageJ software.

### 2.15 Mitochondrial dysfunction assay

The effect of α-KG on PA-induced ferroptosis in SEECs was also confirmed by assessing the mitochondrial dysfunction via analyzing the mitochondrial activities, MMP collapse, and ultrastructural changes (33).

For assessing the mitochondrial activities, SEECs post-drug treatment were fixed and incubated with 200 nM MitoTracker solution (C1049, Beyotime, Shanghai, China) at 37 °C for 30 min. After DAPI counterstaining, the representative MitoTracker staining results were recorded by confocal microscopy, and the staining intensity was quantitatively analyzed using ImageJ software.

For assessing the MMP collapse, SEECs post-drug treatment were incubated with 10 μM JC-1 solution (C2006, Beyotime, Shanghai, China) at 37 °C for 20 min. After microscopic observation, the MMP collapse of each group was analyzed with the red (J-agg)/green (J-mon) fluorescence ratio calculated using ImageJ software.

For assessing ultrastructural changes, SEECs post-drug treatment were harvested and fixed with 2.5% glutaraldehyde (G1102, Solarbio, Beijing, China) followed by post-fixation with 1% OsO_4_, dehydration in ethanol, embedding, and sectioning. The prepared ultrathin sections were stained with uranyl acetate and lead citrate and then visualized using transmission electron microscopy (TEM) by the Shandong Huiyan Scientific Services Co., Ltd. (Qingdao, China) (21).

### 2.16 Biochemical assessment

To confirm the potential effect of α-KG against PA-induced ferroptosis, SEECs post-drug treatment were harvested with the ferroptosis-related biomarker, including superoxide dismutase (SOD), GSH, malondialdehyde (MDA), and iron, detected using commercial assay kits (BC0175 for SOD, BC1175 for GSH, BC0025 for MDA, BC5315 for iron, Solarbio, Beijing, China).

### 2.17 Western blot

To confirm the potential effect of α-KG against PA-induced alterations in the expression levels of apoptosis, histone trimethylation, and ferroptosis related proteins, protein lysates from SEECs post-drug treatment were extracted using RIPA lysis buffer (R0010, Solarbio, Beijing, China), and then quantified using BCA assay (PC0020, Solarbio, Beijing, China) according to the manufacturer’s instructions (33). Protein lysates from each group were separated on 10% SDS-PAGE gels (P1200, Solarbio, Beijing, China) and transferred onto PVDF membranes (IPVH00010, Millipore, Beijing, China). The membranes were blocked with 5% (w/v) non-fat dry milk (D8340, Solarbio, Beijing, China) for 1 h and incubated at 4 °C overnight with primary antibodies. The membranes were further incubated with secondary antibodies at room temperature for 1 h. Protein bands were visualized using enhanced chemiluminescence (ECL, W1001, Promega, Beijing, China) and imaged with a chemiluminescence imaging system. A list of antibodies used is provided in the Supplementary Table 1.

### 2.18 Statistical analysis

Data were expressed as mean ± standard deviation (SD). Statistical significance was determined using SPSS software (IBM, version 19.0) with one-way ANOVA followed by post-hoc LSD or non-parametric Wilcoxon tests based on the data distribution. A *p* value of <0.05 was considered statistically significant.

## 3 Result

### 3.1 Protective effect of α-KG on PA-induced deterioration of SEECs

To investigate the protective role of α-KG in mitigating PA-induced deterioration in SEECs, we first assessed its effect on cell viability following 24 h of PA exposure. As shown in Supplementary Figure 1C, the treatment with α-KG at all tested concentrations (1.5, 3, and 6 mM) effectively improved SEECs viability compared to the PA-treated group (*p* < 0.05). Moreover, LDH activity, which was markedly elevated in the PA-treated group relative to the NC group (Supplementary Figure 1D, *p* < 0.05), was effectively reduced by α-KG treatment. The progressive increase in viability and decline in LDH activity from the PA group to all α-KG-treated groups strongly confirmed the protective effect of α-KG against PA-induced cytotoxicity in SEECs. Given the absence of a significant difference in the viability and LDH activity between the PA + 3 mM α-KG and PA + 6 mM α-KG groups, 3 mM was selected as the optimal concentration of α-KG for subsequent experiments.

**Figure 1:**
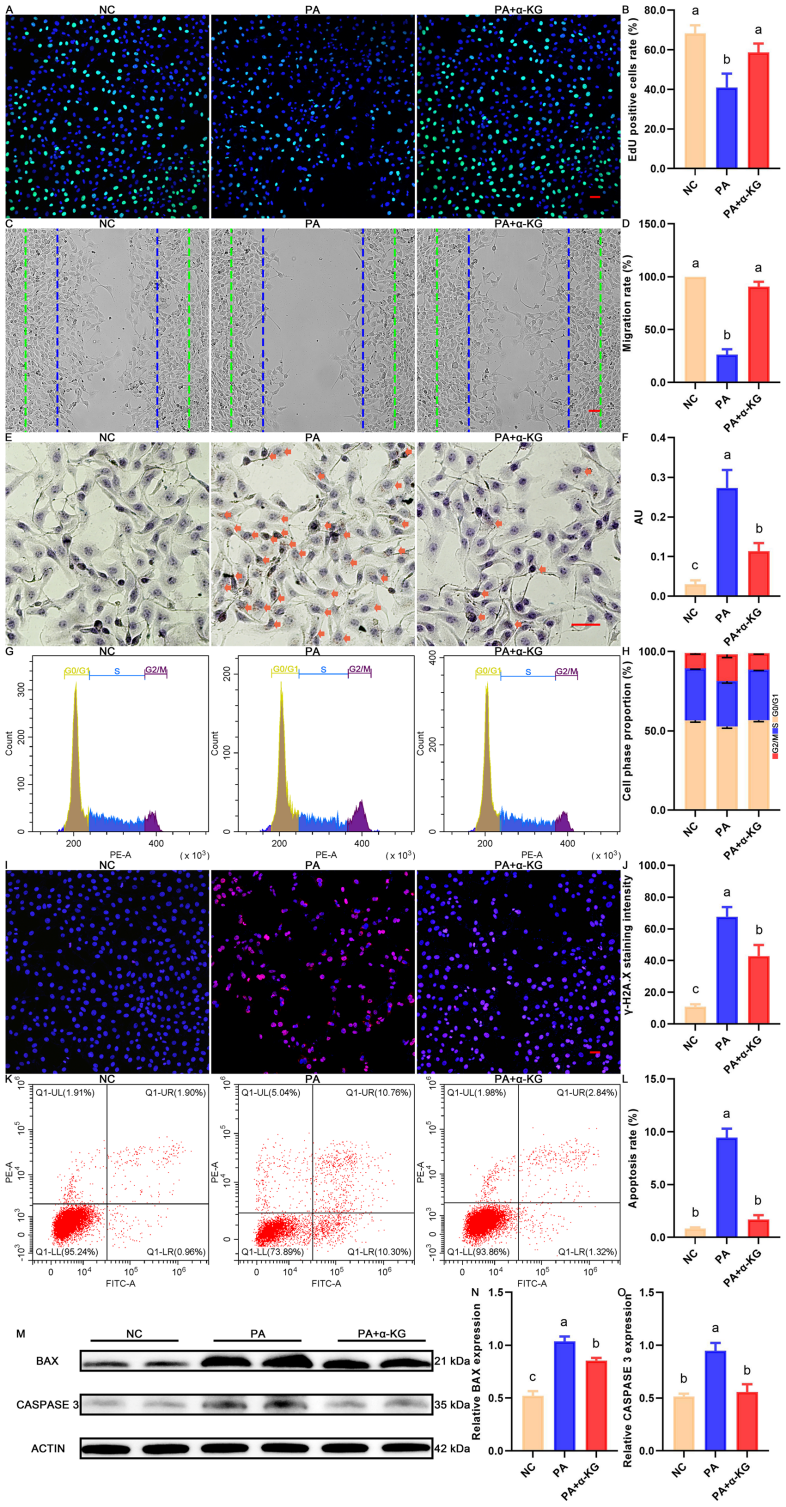
Protective effect of α-KG on PA-induced deterioration of SEECs. **(A)** Representative result of EdU staining. Scale bar = 100 μm. **(B)** Corresponding quantification result of EdU staining. **(C)** Representative result of cell migration. Scale bar = 100 μm. **(D)** Corresponding quantification result of cell migration. **(E)** Representative result of Oil Red O staining. Scale bar = 100 μm. **(F)** Corresponding quantification result of Oil Red O staining. **(G)** Representative results of cell cycle distribution. **(H)** Corresponding quantification result of cell cycle distribution. **(I)** Representative IF staining result of γH2A. X. Scale bar = 100 μm. **(J)** Corresponding quantification result of IF staining of γH2A. X. **(K)** Representative FACS result of Annexin V/PI staining. **(L)** Quantification result of Annexin V/PI staining. **(M)** Representative western blot results of apoptosis-related proteins. **(N)** Quantitative western blot result of BAX. **(O)** Quantitative western blot result of CASPASE 3. Different lowercase letters in the bar graph indicate significant differences between groups (*p* < 0.05).

Consistent with the MTT and LDH findings, EdU incorporation staining further confirmed the protective effect of α-KG on PA-induced proliferation inhibition. The percentage of EdU-positive SEECs was significantly reduced in the PA-treated group compared to the NC group (Figures 1A,B, *p* < 0.05), whereas co-treatment with α-KG significantly restored EdU incorporation, highlighting its ability to counteract PA-induced proliferative deficits. In addition to impairing proliferation, PA exposure markedly disrupted key cellular functions in SEECs, as evidenced by decreased migration potential (Figures 1C,D, *p* < 0.05) and increased lipid accumulation (Figures 1E,F, *p* < 0.05). Furthermore, PA treatment induced significant perturbations in the cell cycle (Figures 1G,H, and Supplementary Figure 2), elevated DNA damage (Figures 1I,J, *p* < 0.05), and increased apoptosis (Figures 1K,L, *p* < 0.05). As anticipated, co-treatment with α-KG effectively alleviated these PA-induced cellular impairments, as indicated by enhanced migratory capacity, ameliorated cell cycle progression, and reduced lipid accumulation, DNA damage, and apoptosis in the PA + α-KG group compared to the PA group. At the molecular level, these protective effects were corroborated by alterations in the expression of apoptosis-related proteins. Western blot analysis revealed significant differential expression of BAX and Caspase 3 among the NC, PA, and PA + α-KG groups (Figures 1M–O, *p* < 0.05), further supporting the role of α-KG in mitigating PA-induced apoptotic signaling.

Collectively, these findings provide compelling evidence that α-KG confers protective effects against PA-induced cellular dysfunction in SEECs by ameliorating PA-mediated impairments in cell survival, proliferation, migration, cell cycle regulation, DNA integrity, and apoptosis.

### 3.2 Ameliorative effect of α-KG on the PA-induced histone trimethylation alteration of SEECs

**Figure 2:**
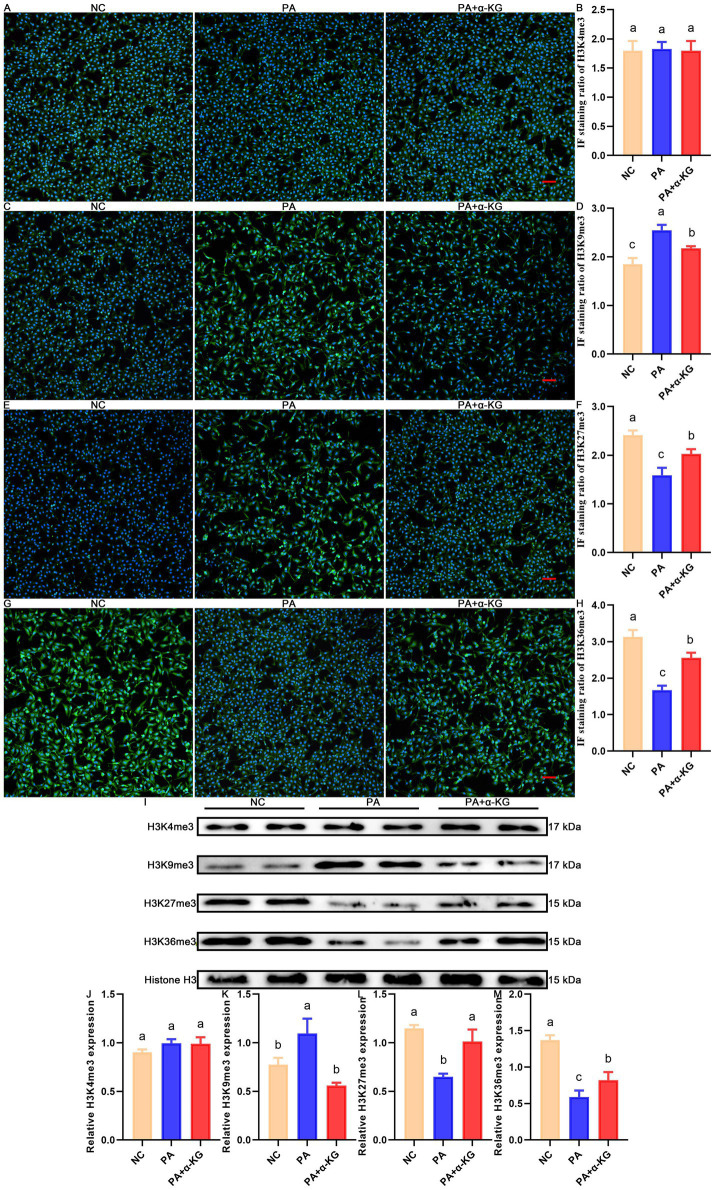
Ameliorative effect of α-KG on the PA-induced histone trimethylation alteration of SEECs. **(A)** Representative IF staining result of H3K4me3. Scale bar = 100 μm. **(B)** Corresponding quantification result of IF staining of H3K4me3. **(C)** Representative IF staining result of H3K9me3. Scale bar = 100 μm. **(D)** Corresponding quantification result of IF staining of H3K9me3. **(E)** Representative IF staining result of H3K27me3. Scale bar = 100 μm. **(F)** Corresponding quantification result of IF staining of H3K27me3. **(G)** Representative IF staining result of H3K36me3. Scale bar = 100 μm. **(H)** Corresponding quantification result of IF staining of H3K36me3. **(I)** Representative western blot results of histone trimethylation-related proteins. **(J)** Quantitative western blot result of H3K4me3. **(K)** Quantitative western blot result of H3K9me3. **(L)** Quantitative western blot result of H3K27me3. **(M)** Quantitative western blot result of H3K36me3. Different lowercase letters in the bar graph indicate significant differences between groups (*p* < 0.05).

Considering the adverse effect of PA on histone trimethylation, the expression pattern and levels of H3K4me3, H3K9me3, H3K27me3, and H3K36me3 were analyzed by IF staining and Western blot, with the results shown in Figure 2.

Compared to the NC group, the PA exposure resulted in a significant increase in the IF staining intensity of H3K9me3 (Figures 2C,D, *p* < 0.05). Additionally, the IF staining intensity of H3K27me3 (Figures 2E,F, *p* < 0.05) and H3K36me3 (Figures 2G,H, *p* < 0.05) was notably reduced in PA-treated SEECs, while no significant change was observed in the IF staining intensity of H3K4me3 between the NC and PA groups (Figures 2A,B, *p* > 0.05). Moreover, the Western blot analysis confirmed these IF staining results, showing a significant upregulation of H3K9me3 levels from the NC group to the PA group (Figures 2I,K, *p* < 0.05). In contrast, the level of H3K27me3 (Figures 2I,L, *p* < 0.05) and H3K36me3 (Figures 2I,M, *p* < 0.05) was markedly decreased from the NC group to the PA group (Figure 2, *p* < 0.05), while no significant change was observed in H3K4me3 levels between the NC and PA groups (Figures 2I,J, *p* > 0.05).

Upon α-KG co-supplementation, the PA-induced accumulation of H3K9me3 was significantly reduced, and the level of H3K27me3 and H3K36me3 was partially restored, as confirmed by both IF staining and Western blot. These findings promisingly suggest that α-KG mitigates PA-induced histone trimethylation alterations.

### 3.3 Mitigating effect of α-KG on PA-induced transcriptomic dysregulation of SEECs

To further elucidate the mechanisms underlying the protective effects of α-KG against PA-induced deterioration of SEECs, transcriptomic analysis of SEECs post-drug treatment was performed. Validation of the transcriptomic data was conducted through Pearson correlation analysis (Supplementary Figure 3A), principal component analysis (PCA, Supplementary Figure 3B), and gene expression profiling (Supplementary Figure 3C). These analyses revealed significant differences across the NC, PA, and PA + α-KG groups, providing a foundation for subsequent in-depth result analysis.

**Figure 3:**
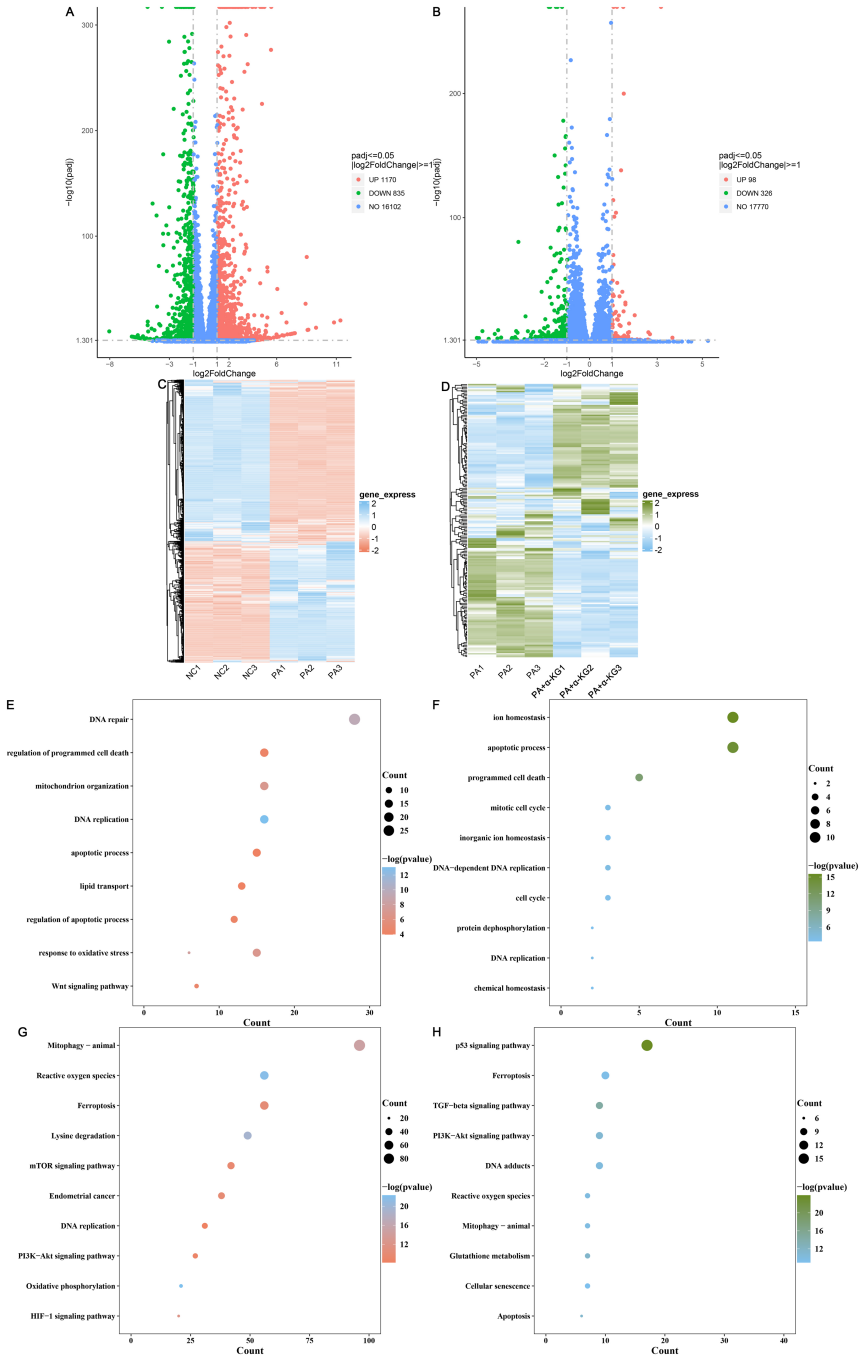
Mitigating effect of α-KG on PA-induced transcriptomic dysregulation of SEECs. **(A)** Volcano plots of DEGs between the NC and PA groups. **(B)** Volcano plots of DEGs between the α-KG and PA groups. **(C)** Heat map of DEGs between the NC and PA groups. **(D)** Heat map of DEGs between the PA + α-KG and PA groups. **(E)** Biological process of GO enrichment analysis between the NC and PA groups. **(F)** Biological process of GO enrichment analysis between the PA + α-KG and PA groups. **(G)** KEGG pathway enrichment analysis between the NC and PA groups. **(H)** KEGG pathway enrichment analysis between the PA + α-KG and PA groups.

A total of 12,515 transcripts were identified across the NC, PA, and PA + α-KG groups. Differential expression analysis revealed that PA treatment led to significant transcriptomic alterations, with 2005 DEGs, comprising 1,170 upregulated and 835 downregulated, compared to the NC group (Figure 3A). In contrast, co-treatment with α-KG resulted in 424 DEGs relative to the PA group, with 98 upregulated and 326 downregulated (Figure 3B). Hierarchical clustering based on the heatmap analysis demonstrated distinct transcriptomic profiles between the NC and PA groups (Figure 3C), as well as between the PA and PA + α-KG groups (Figure 3D), underscoring the profound transcriptional reprogramming induced by PA exposure and the substantial restorative effects of α-KG.

GO enrichment analysis between the NC and PA groups revealed significant enrichment in biological processes related to DNA replication, DNA repair, response to oxidative stress, mitochondrion organization, response to oxidative stress, Wnt signaling pathway, regulation of apoptotic process, regulation of programmed cell death, lipid transport, and apoptotic process (Figure 3E). Additionally, KEGG pathway analysis between the NC and PA groups indicated that PA-induced SEECs deterioration was closely associated with pathways regulating mitophagy-animal, chemical carcinogenesis-reactive oxygen species, ferroptosis, lysine degradation, mTOR signaling pathway, endometrial cancer, DNA replication, PI3K-Akt signaling pathway, oxidative phosphorylation, and HIF-1 signaling pathway (Figure 3G).

Conversely, GO enrichment analysis between the PA and PA + α-KG groups demonstrated that co-treatment with α-KG modulated biological processes, including ion homeostasis, apoptotic process, programmed cell death, DNA-dependent DNA replication, mitotic cell cycle, DNA replication, cell cycle, inorganic ion homeostasis, chemical homeostasis, and protein dephosphorylation (Figure 3F). KEGG pathway analysis further confirmed that α-KG exerted protective effects against PA-induced SEECs deterioration by modulating pathways involved in the p53 signaling pathway, TGF-beta signaling pathway, glutathione metabolism, apoptosis, PI3K-Akt signaling pathway, chemical carcinogenesis-DNA adducts, chemical carcinogenesis-reactive oxygen species, mitophagy-animal, ferroptosis, and cellular senescence (Figure 3H). These findings highlight the critical role of α-KG in mitigating PA-induced transcriptional dysregulation in SEECs.

### 3.4 Alleviative effect of α-KG on PA-induced metabolic perturbations of SEECs

To further investigate the ameliorative effects of α-KG on PA-induced metabolic disturbances in SEECs, we conducted an untargeted metabolomics analysis. Following quality control, the results of PCA and Partial Least Squares Discriminant Analysis (PLS-DA) revealed significant metabolic differences across the NC, PA, and PA + α-KG groups (Supplementary Figure 4), providing a solid foundation for subsequent in-depth analysis.

**Figure 4:**
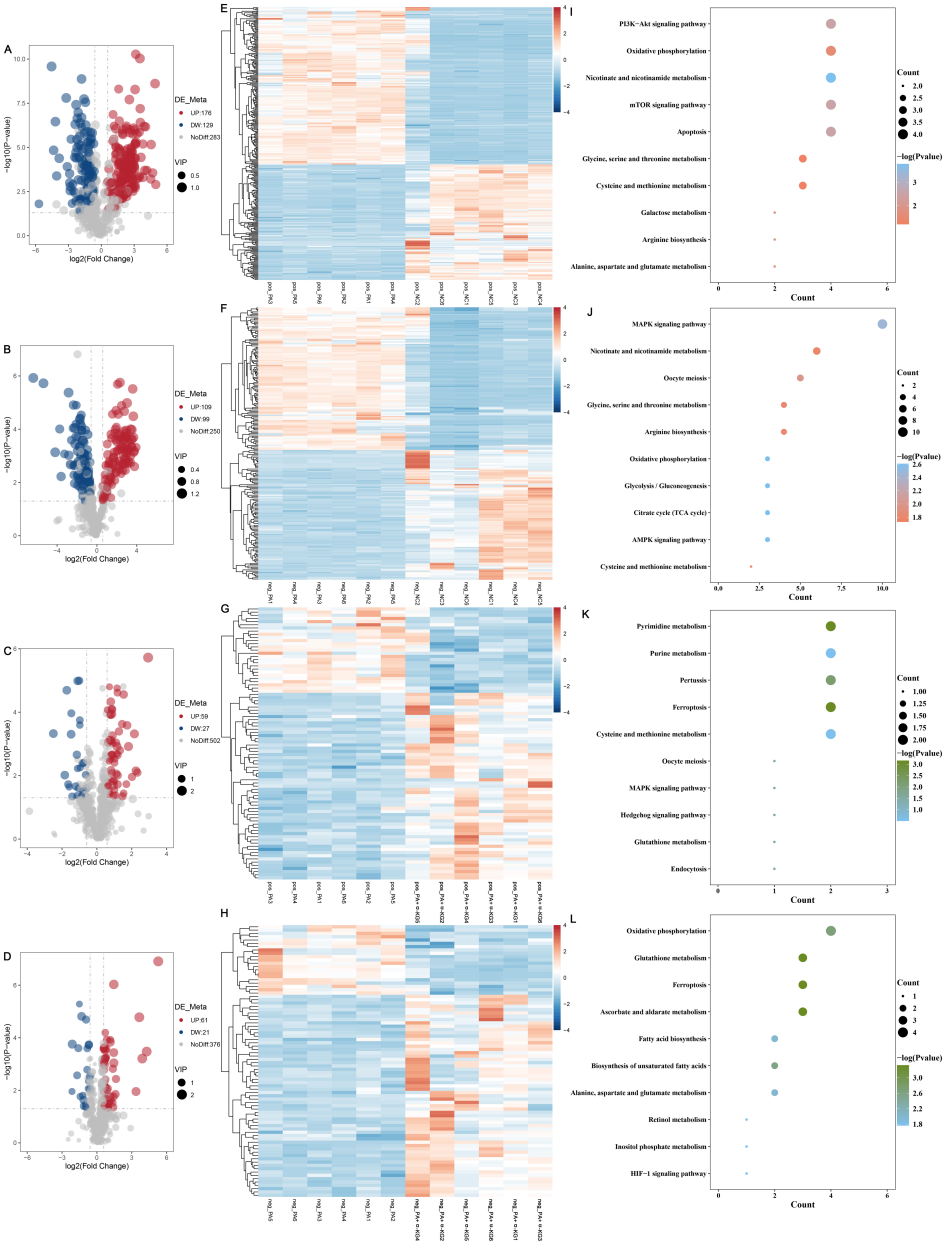
Alleviative effect of α-KG on PA-induced metabolic perturbations of SEECs. **(A)** Volcano plots of differential metabolites in positive ion mode between the NC and PA groups. **(B)** Volcano plots of differential metabolites in negative ion mode between the NC and PA groups. **(C)** Volcano plots of differential metabolites in positive ion mode between the PA + α-KG and PA groups. **(D)** Volcano plots of differential metabolites in negative ion mode between the PA + α-KG and PA groups. **(E)** Heat map of differential metabolites in positive ion mode between the NC and PA groups. **(F)** Heat map of differential metabolites in positive ion mode between the NC and PA groups. **(G)** Heat map of differential metabolites in positive ion mode between the PA + α-KG and PA groups. **(H)** Heat map of differential metabolites in positive ion mode between the PA + α-KG and PA groups. **(I)** KEGG pathway enrichment analysis of differential metabolites in positive ion mode between the NC and PA groups. **(J)** KEGG pathway enrichment analysis of differential metabolites in negative ion mode between the NC and PA groups. **(K)** KEGG pathway enrichment analysis of differential metabolites in positive ion mode between the PA + α-KG and PA groups. **(L)** KEGG pathway enrichment analysis of differential metabolites in negative ion mode between the PA + α-KG and PA groups.

In positive ion mode, a total of 588 metabolites were identified between the PA and NC groups, among which 176 were significantly upregulated and 129 were significantly downregulated (Figure 4A). Similarly, in negative ion mode, 458 metabolites were identified, including 109 significantly upregulated and 99 significantly downregulated metabolites (Figure 4B). Heatmap analysis further confirmed distinct metabolic profiles between the NC and PA groups in both positive and negative ion modes (Figures 4E,F).

To elucidate the biological relevance of these metabolic alterations, all identified metabolites were systematically annotated, and pathway enrichment analysis was conducted using the KEGG database. The results indicated that, in positive ion mode, the differentially abundant metabolites between the PA and NC groups were predominantly involved in key metabolic pathways, including nicotinate and nicotinamide metabolism, mTOR signaling pathway, PI3K-Akt signaling pathway, apoptosis, arginine biosynthesis, alanine, aspartate and glutamate metabolism, galactose metabolism, oxidative phosphorylation, glycine, serine and threonine metabolism, and cysteine and methionine metabolism (Figure 4I). Similarly, KEGG pathway enrichment analysis in negative ion mode (Figure 4J) revealed that the identified metabolites between the PA and NC groups were significantly associated with the citrate cycle (TCA cycle), oxidative phosphorylation, AMPK signaling pathway, glycolysis/gluconeogenesis, MAPK signaling pathway, oocyte meiosis, arginine biosynthesis, glycine, serine and threonine metabolism, nicotinate and nicotinamide metabolism, and cysteine and methionine metabolism.

In contrast, in the PA + α-KG group, metabolomic profiling revealed substantial shifts in metabolic composition compared to the PA group. Specifically, in positive ion mode, 588 metabolites were identified, with 59 significantly upregulated and 27 significantly downregulated (Figure 4C). In negative ion mode, 458 metabolites were detected, including 61 significantly upregulated and 21 significantly downregulated (Figure 4D). Heatmap analysis consistently confirmed distinct metabolic profiles between the PA + α-KG and PA groups, further highlighting the regulatory impact of α-KG on PA-induced metabolic disturbances (Figures 4G,H).

KEGG pathway enrichment analysis of differentially abundant metabolites between the PA + α-KG and PA groups revealed significant associations with metabolic pathways, including purine metabolism, cysteine and methionine metabolism, glutathione metabolism, MAPK signaling pathway, oocyte meiosis, endocytosis, hedgehog signaling pathway, pertussis, ferroptosis, and pyrimidine metabolism (Figure 4K). Similarly, in negative ion mode, co-treatment with α-KG influenced pathways involved in ascorbate and aldarate metabolism, glutathione metabolism, ferroptosis, oxidative phosphorylation, biosynthesis of unsaturated fatty acids, fatty acid biosynthesis, alanine, aspartate and glutamate metabolism, inositol phosphate metabolism, retinol metabolism, and HIF-1 signaling pathway (Figure 4L).

These findings collectively suggest that α-KG effectively mitigates PA-induced metabolic perturbations in SEECs by modulating key metabolic pathways, restoring cellular homeostasis, and counteracting PA-associated metabolic dysregulation.

### 3.5 Ameliorative effect of α-KG on PA-induced ferroptosis in SEECs

Given the transcriptomic and metabolomic evidence of ferroptosis, we hypothesized that the protective effect of α-KG in counteracting PA-induced deterioration of SEECs was closely linked to the regulation of ferroptosis. To test this hypothesis, we evaluated the impact of α-KG on PA-induced ferroptosis in SEECs.

**Figure 5:**
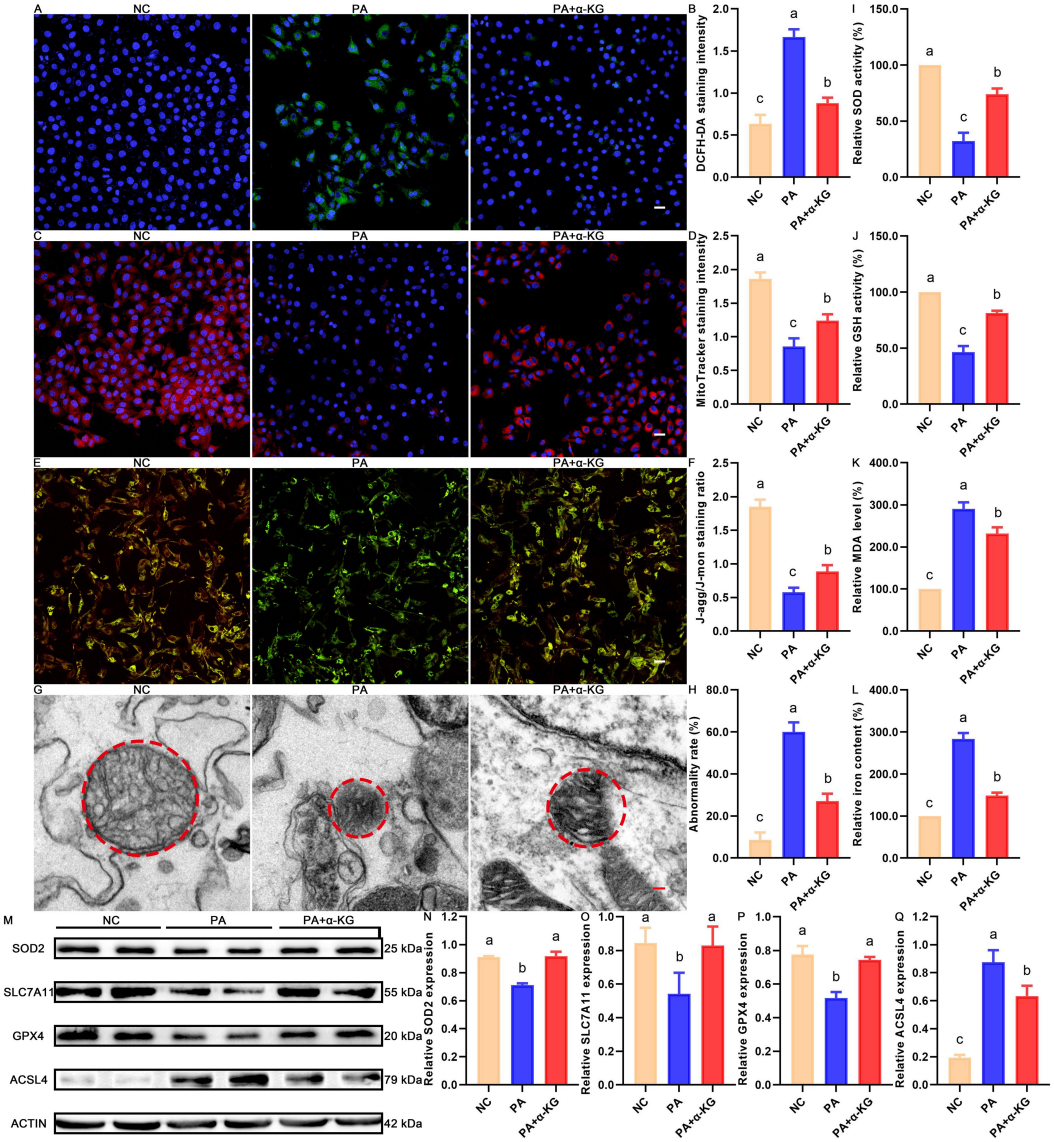
Ameliorative effect of α-KG on PA-induced ferroptosis in SEECs. **(A)** Representative result of DCFH-DA staining. Scale bar = 100 μm. **(B)** Quantitative result of DCFH-DA staining. **(C)** Representative result of MitoTracker staining. Scale bar = 100 μm. **(D)** Quantitative result of MitoTracker staining. **(E)** Representative result of JC-1 staining. Scale bar = 100 μm. **(F)** Quantitative result of JC-1 staining. **(G)** Representative TEM results. Scale bar = 200 nm. **(H)** Quantitative result of mitochondrial abnormality. **(I)** Quantitative results of SOD activity. **(J)** Quantitative results of GSH activity. **(K)** Quantitative results of MDA activity. **(L)** Quantitative results of iron content. **(M)** Representative western blot result of ferroptosis-related proteins. **(N)** Representative western blot result of SOD2. **(O)** Representative western blot result of SLC7A11. **(P)** Representative western blot result of GPX4. **(Q)** Representative western blot result of ACSL4. Different lowercase letters in the bar graph indicate significant differences between groups (*p* < 0.05).

As shown in Figures 5A,B, the fluorescence staining intensity of DCFH-DA, a marker of ROS production, was significantly elevated in the PA group compared to the NC group (*p* < 0.05), confirming that PA exposure promotes ROS accumulation in SEECs. Notably, co-treatment with α-KG effectively mitigated PA-induced ROS overproduction, as evidenced by the significant reduction in DCFH-DA fluorescence in the PA + α-KG group relative to the PA group (*p* < 0.05).

In addition, PA exposure resulted in a marked reduction in MitoTracker fluorescence intensity (Figures 5C,D, *p* < 0.05) and MMP collapse, as indicated by the altered J-agg/J-mon ratio (Figures 5E,F, *p* < 0.05). Coupled with mitochondrial structural abnormalities, such as cristae disruption and membrane rupture (Figures 5G,H, *p* < 0.05), these findings collectively confirm that PA induces mitochondrial dysfunctions in SEECs. In contrast, co-treatment with α-KG effectively ameliorated these mitochondrial impairments, restoring MitoTracker fluorescence intensity (*p* < 0.05), preserving MMP stability (*p* < 0.05), and mitigating mitochondrial damage in SEECs (*p* < 0.05), thereby underscoring its protective role in maintaining mitochondrial integrity and function.

Furthermore, as shown in Figures 5I,J, SOD and GSH activities were significantly reduced in PA-treated SEECs compared to the NC group (*p* < 0.05). Concurrently, lipid peroxidation, reflected by increased MDA levels, was significantly elevated in the PA group (Figure 5K, *p* < 0.05). Along with the observed increase in intracellular iron levels (Figure 5L, *p* < 0.05), these findings provide strong evidence of PA-induced ferroptosis in SEECs. However, co-treatment with α-KG significantly restored SOD and GSH activities while reducing MDA levels and iron accumulation in SEECs compared to the PA group (*p* < 0.05).

Taken together, these results, in conjunction with the different expression profiles of ferroptosis-related proteins including SOD2 (Figures 5M,N), SLC7A11 (Figures 5M,O), GPX4 (Figures 5M,P), and ACSL 4 (Figures 5M,Q) across the NC, PA, and PA + α-KG groups, provide compelling evidence that α-KG exerts a protective role in mitigating PA-induced ferroptosis in SEECs.

## 4 Discussion

Consistent with previous reports (18–21), our study demonstrates for the first time that PA exposure induces the deterioration of SEECs by reducing cellular viability, proliferation, and migration and by inducing cell cycle arrest, DNA damage, apoptosis, aberrant histone trimethylation, and ferroptosis. Notably, these detrimental effects were significantly ameliorated by the co-treatment with α-KG, highlighting its potential as a metabolic and epigenetic modulator in lipid-induced cellular dysfunction.

Histone methylation, a fundamental epigenetic regulatory mechanism, predominantly occurs on lysine (K) and arginine (R) residues of histones H3 and H4. This modification plays a critical role in the regulation of gene expression, chromatin remodeling, and genomic integrity processes, which are integral to cell fate determination and developmental programming (36). Lysine residues may undergo mono-, di-, or tri-methylation, while arginine residues can be mono-methylated or di-methylated in either symmetric or asymmetric configurations (36). These marks are dynamically controlled by opposing enzymatic activities: histone methyltransferases (HMTs), such as lysine methyltransferases (KMTs) and protein arginine methyltransferases (PRMTs), serve as “writers” by adding methyl groups, whereas histone demethylases (HDMs), including lysine-specific demethylases (LSDs) and JMJDs, function as “erasers” by removing them (36).

Among the diverse repertoire of histone modifications, trimethylation of specific histone H3 lysine residues-notably H3K4me3, H3K9me3, H3K27me3, and H3K36me3-has received considerable attention due to its prominent role in epigenetic regulation of gene expression (37–41). H3K4me3 and H3K36me3 are typically associated with transcriptionally active chromatin and are enriched at promoter and gene body regions, respectively, facilitating transcriptional activation. Conversely, H3K9me3 and H3K27me3 are hallmarks of transcriptional repression and chromatin condensation, linked to heterochromatin formation and gene silencing. These trimethylation marks operate within a tightly interconnected network of covalent histone modifications, coordinating critical cellular processes such as transcriptional regulation, DNA replication, repair, and cell cycle progression. Altered patterns in the expression of these epigenetic marks have been implicated in a variety of reproductive pathologies, including endometriosis, endometrial carcinomas, pregnancy complications, and infertility (42–47).

Consistent with prior findings (20), our results demonstrate that PA exposure leads to a significant upregulation of H3K9me3, which is associated with transcriptional repression. In contrast, H3K27me3 and H3K36me3 levels were markedly reduced, suggesting impaired transcriptional capacity. No significant changes were observed in H3K4me3, indicating selective sensitivity of specific trimethylation marks to PA-induced stress. Collectively, these results highlight the detrimental role of PA in disrupting histone methylation homeostasis, further supporting its contribution to epigenetic dysregulation. Importantly, although H3K27me3 is classically recognized as a transcriptional repressive mark, its reduction under PA exposure does not necessarily translate into beneficial transcriptional activation. Instead, loss of H3K27me3 may precipitate selective and maladaptive de-repression of stress-responsive, inflammatory, or pro-ferroptotic gene programs, thereby aggravating cellular dysfunction (48–50). Mechanistic evidence supports this interpretation. For example, Xu et al. (51) demonstrated that PA treatment in cardiomyocytes significantly reduces H3K27me3 levels, concomitantly upregulated ACSL4 expression, and promoted ferroptosis, whereas pharmacological inhibition of KDM6A/B with GSK-J4 preserved H3K27me3 and alleviated lipotoxic injury.

In line with this, Schuldt et al. (52) reported that palmitate-challenged periodontal ligament fibroblasts exhibited altered H3K27me3 enrichment at the IL-10 promoter, resulting in reduced IL-10 expression and a hyperinflammatory phenotype, highlighting that loss of repressive marks can also impair anti-inflammatory pathways in a context-dependent manner. Furthermore, PA has been shown to activate H3K27 demethylases such as JMJD3/KDM6B through TLR4-dependent signaling, thereby erasing H3K27me3 from NF-κB target promoters and driving inflammatory transcription; inhibition of these demethylases by GSK-J4 effectively attenuated PA-induced inflammation both *in vitro* and *in vivo* (53). Collectively, these findings indicate that the reduction of H3K27me3 observed in our PA-treated SEECs does not represent a uniform release of transcriptional repression, but rather a selective and pathological reprogramming of the chromatin landscape. Such reprogramming may aberrantly activate inflammatory, ferroptotic, and pro-apoptotic pathways while simultaneously suppressing protective responses, ultimately contributing to the deleterious phenotype. The concomitant increase in H3K9me3 in our study further underscores the disruption of the balance among repressive histone marks, consistent with a global dysregulation of chromatin homeostasis under lipotoxic stress. Despite these insights, the precise molecular mechanisms by which PA orchestrates the differential regulation of histone trimethylation marks, including H3K4me3, H3K9me3, H3K27me3, and H3K36me3, remain to be fully elucidated. Our study demonstrates clear alterations in these modifications under lipotoxic stress; however, it does not comprehensively address the upstream signaling pathways, the specific histone-modifying enzymes involved, or the gene networks directly affected by these changes. Future studies integrating chromatin immunoprecipitation sequencing, enzyme activity assays, and transcriptome-wide analyses will be necessary to delineate the precise regulatory circuitry to provide a more comprehensive understanding of how PA-induced epigenetic dysregulation contributes to lipotoxic injury and reproductive dysfunction.

Notably, our findings revealed that α-KG conferred cellular protection by mitigating PA-induced disruptions in histone trimethylations. Beyond its established role as a central intermediate in the TCA cycle, α-KG also functions as a pivotal epigenetic cofactor. It facilitates histone demethylation through α-KG/Fe(II)-dependent dioxygenases, including JHDMs. In our study, α-KG supplementation selectively reversed PA-induced accumulation of H3K9me3 while partially restoring the expression levels of H3K27me3 and H3K36me3. These results suggest that α-KG remodels chromatin structure and transcriptional programs by modulating histone methylation, thereby preserving SEECs function under lipotoxic stress. However, the precise molecular mechanisms underlying α-KG’s epigenetic modulation in reproductive pathologies warrant further investigation.

Moreover, we further demonstrated that α-KG exerts protective effects against PA-induced dysfunction in SEECs and that this protective effect is closely associated with the inhibition of ferroptosis. Ferroptosis, a recently recognized form of programmed cell death, is characterized by iron-dependent lipid peroxidation, which distinguishes it from other forms of cell death. It is influenced by free radicals and lipid metabolism (54). A hallmark of ferroptosis is the overwhelming peroxidation of polyunsaturated fatty acids (PUFAs) within membrane phospholipids, eventually leading to plasma membrane rupture (55).

Since its identification in 2012, ferroptosis has garnered significant attention due to its involvement in a wide array of physiological and pathological conditions, including autoimmune disorders, cardiovascular diseases, cancer malignancies, genetic disorders, metabolic disorders, chronic inflammatory diseases, musculoskeletal diseases, and neurodegenerative diseases (54, 56). Notably, germline cells are particularly susceptible to ferroptosis, especially under pathological conditions, due to their inherent vulnerability to oxidative stress and dysregulation of iron metabolism (57–59). Extensive research has highlighted the role of ferroptosis in various reproductive disorders, such as endometrial fibrosis, endometriosis, polycystic ovary syndrome (PCOS)-related infertility, and tubal factor infertility (57, 60–62). As a result, considerable interest has developed in identifying pharmacological strategies to modulate ferroptosis. Numerous drugs and biologically active agents have been identified as either inducers or inhibitors of ferroptosis, offering therapeutic potentials (63).

Recent studies have further established PA as a potent inducer of ferroptosis across various disease models. Wang et al. (64) demonstrated that PA downregulates GPX4 and heat shock factor 1 (HSF1), thus triggering ferroptosis in cardiomyocytes. Similarly, Zhu et al. (65) reported that PA promotes ceramide synthesis and inhibits GPX4, driving ferroptosis in pancreatic β-cells-a finding that is consistent with studies by Feng et al. (66) and Du et al. (67). In hepatocytes, Liao et al. (68) identified upregulation of Cytochrome P450-2E1 (CYP2E1) and nuclear translocation of sterol regulatory element-binding protein 1 (SREBP1) as critical mechanisms underlying PA-induced ferroptosis, contributing to metabolic dysfunction-associated fatty liver disease (MAFLD). Moreover, Guan et al. (41) found that PA suppresses SLC7A11 and GPX4 in vascular endothelial cells, a finding further corroborated by studies from Xie et al. (69, 70), Tan et al. (71), and Lan et al. (72). Collectively, these studies position PA as a universal inducer of ferroptosis across diverse cell types, underscoring its significant pathophysiological role and therapeutic potential. In addition to its direct ferroptotic effects, PA could be metabolized into lysophosphatidic acid (LPA) via glycerol-3-phosphate acyltransferase 1 (GPAT1), which then generates phosphatidic acid. This metabolite activates protein kinase C *ζ* (PKCζ), modulating the activity of phosphatidylcholine-binding protein 1 (PEBP1) and the 15-lipoxygenase (15-LO) complex, thereby amplifying the peroxidation of PUFAs and enhancing ferroptosis (73). Consistent with previous studies, our findings demonstrate that PA significantly increases intracellular ROS levels, induces mitochondrial dysfunction, elevates MDA and free iron concentrations, depletes SOD and GSH activities, downregulates the ferroptosis-inhibitory proteins SOD2, SLC7A11, and GPX4, and upregulates the ferroptosis executioner protein ASCL4 in SEECs. These results further underscore the pivotal role of PA in activating ferroptosis, with far-reaching implications for disease pathogenesis and potential therapeutic strategies.

Given the critical involvement of ferroptosis in the onset and progression of various diseases, small-molecule inhibitors and inducers targeting this process have been increasingly explored. These compounds have shown promise in modulating ferroptosis, offering potential therapeutic avenues for treating a wide range of disorders. In addition, ferroptosis inhibition has been proven with the effective benefit profiles of the advancements in reproductive biotechnologies in small ruminants, as well as the enhancement of the breeding programs (74–77). In our current study, we found that the protective effects of α-KG against PA-induced cellular dysfunction in SEECs were positively related to the inhibition of ferroptosis. Emerging evidence suggests that α-KG plays a protective role in lipid metabolism and defends against lipid peroxidation damage (78). In 2024, Yu et al. (79) demonstrated that α-KG promotes mitophagy, inhibits ferroptosis, and mitigates myocardial cell injury through the NAD⁺-SIRT1 signaling pathway. In alignment with our findings, He et al. (78) reported that α-KG markedly attenuates osteoarthritis progression by reducing cartilage matrix degradation and apoptosis, suppressing ROS, MDA, and ferrous ion accumulation, enhancing SOD activity and the GSH/GSSG ratio, preserving MMP stability, and upregulating ETV4, SLC7A11, and GPX4. Contrastingly, other studies have reported that α-KG may promote ferroptosis in cancer cells. For instance, α-KG has been shown to mimic the death-promoting effect of glutamine, supporting the role of glutaminolysis in ferroptotic processes (80, 81). In this context, α-KG can be utilized by dihydrolipoamide dehydrogenase (DLD) in the mitochondria to generate ROS or converted into cytosolic acetyl-CoA, fueling fatty acid synthesis, increasing DNA oxidation damage-mediated TP53 expression, and contributing to ferroptosis (82–84). α-KG also downregulated SLC7A11, elevated oxidative stress, and induced DNA damage of liver cancer cells (27). Additionally, Wang et al. found that the metabolic conversion of α-KG into (D)-2-hydroxyglutarate potentiates erastin-induced ferroptosis. Moreover, Hu et al. (85) reported that reduced expression of branched-chain amino acid transaminase 1 (BCAT1) impairs GPX4 transcription via a rapid-response metabolism–epigenetics axis involving α-KG accumulation, loss of H3K9me3 modification, and upregulation of early growth response protein 1 (EGR1), thereby sensitizing mesenchymal stromal cells to ferroptosis (86).

These seemingly contradictory observations may be attributed to variations in cell type, metabolic state, and species-specific responses, as well as the inherently multifaceted and context-dependent functions of α-KG. Under physiological conditions, α-KG plays a pivotal role in maintaining redox homeostasis by enhancing cellular antioxidant capacity. It functions as a scavenger of ROS and promotes the biosynthesis of GSH, thereby protecting cells from oxidative damage and preserving mitochondrial integrity. In contrast, within the oncogenic microenvironment, α-KG exhibits a paradoxical behavior. Rather than mitigating oxidative stress, α-KG amplifies ROS accumulation, disrupts redox equilibrium, and exceeds the oxidative stress tolerance of tumor cells. This redox imbalance contributes to mitochondrial dysfunction and promotes both ferroptotic and apoptotic cell death (27). These context-dependent effects highlight the dualistic nature of α-KG as both a cytoprotective and cytotoxic metabolite. Its function appears to be finely tuned by cell-specific metabolic reprogramming, subcellular localization, and the broader pathological milieu. Given its central role in cellular metabolism, epigenetic regulation, and oxidative stress response, further mechanistic investigations are warranted to unravel the bidirectional role of α-KG in ferroptosis and to explore its therapeutic potential across diverse disease models.

Nevertheless, our findings underscore the need for further in-depth investigations to elucidate the protective effects of α-KG against PA-induced deterioration in SEECs, particularly through the inhibition of ferroptosis. Moreover, these results highlight the therapeutic potential of α-KG in mitigating PA-induced metabolic disturbances in SEECs, paving the way for its future applications.

## Data Availability

The raw sequence data reported in this paper have been deposited in the Genome Sequence Archive (Genomics, Proteomics & Bioinformatics 2025) in National Genomics Data Center (Nucleic Acids Res 2025), China National Center for Bioinformation/Beijing Institute of Genomics, Chinese Academy of Sciences (GS accession number: CRA032418), https://ngdc.cncb.ac.cn/gsa.
