# Supplementary material for: Protective effects of α-ketoglutaric acid on palmitic acid-induced deterioration in sheep endometrial epithelial cells via ferroptosis inhibition
Source: Front Vet Sci. 2025 Nov 12;12:1617348. doi: 10.3389/fvets.2025.1617348 (PMC12658361; doi:10.3389/fvets.2025.1617348)
Supplement: Supplementary file 1 [file Data_Sheet_1.docx]

**Supplementation Figures and tables legends**

**Supplementation Figure 1. Identification of SEECs and Protective Effect of** **α-KG on PA-Induced Viability Deficits in SEECs**

A: Representative IF staining of CK18. Scale bar=100 μm. B: Representative IF staining of Vimentin. Scale bar=100 μm. C: Quantitative viability of SEECs following 24 h of treatment with PA and varying concentrations of α-KG. D: Quantification of LDH release from SEECs following 24 h of treatment with PA and varying concentrations of α-KG. Different lowercase letters in the bar graph indicate significant differences between groups (P < 0.05).

**Supplementation Figure 2. Protective Effect of α-KG on PA-Induced Cell Cycle Disruptions of SEECs**

A: Quantitative analysis of the G1/G0 phase proportion. B: Quantitative analysis of the S phase proportion. C: Quantitative analysis of the G2/M phase proportion. Different lowercase letters in the bar graph indicate significant differences between groups (P < 0.05).

**Supplementation Figure 3. Pearson Correlation, Principal Component Analysis (PCA), and Gene Expression Profiling of Transcriptomic Data**

**A: Pearson correlation analysis between experimental groups. B: Principal component analysis (PCA) of transcriptomic data. C: Distribution of gene expression patterns across experimental conditions.**

**Supplementary Figure 4. PCA and Partial Least Squares Discriminant Analysis (PLS-DA) of Metabolomic Data**

A: PCA in positive ion mode comparing NC and PA groups. B: PLS-DA in positive ion mode comparing NC and PA groups. C: PCA in negative ion mode comparing NC and PA groups. D: PLS-DA in negative ion mode comparing NC and PA groups. E: PCA in positive ion mode comparing PA+α-KG and PA groups. F: PLS-DA in positive ion mode comparing PA+α-KG and PA groups. G: PCA in negative ion mode comparing PA+α-KG and PA groups. H: PLS-DA in negative ion mode comparing PA+α-KG and PA groups.

**Supplementation Table 1. Antibodies utilized in this study**

**Figures and table**

**Supplementation Figure 1. Identification of SEECs and Protective Effect of α-KG on PA-Induced Viability Deficits in SEECs**


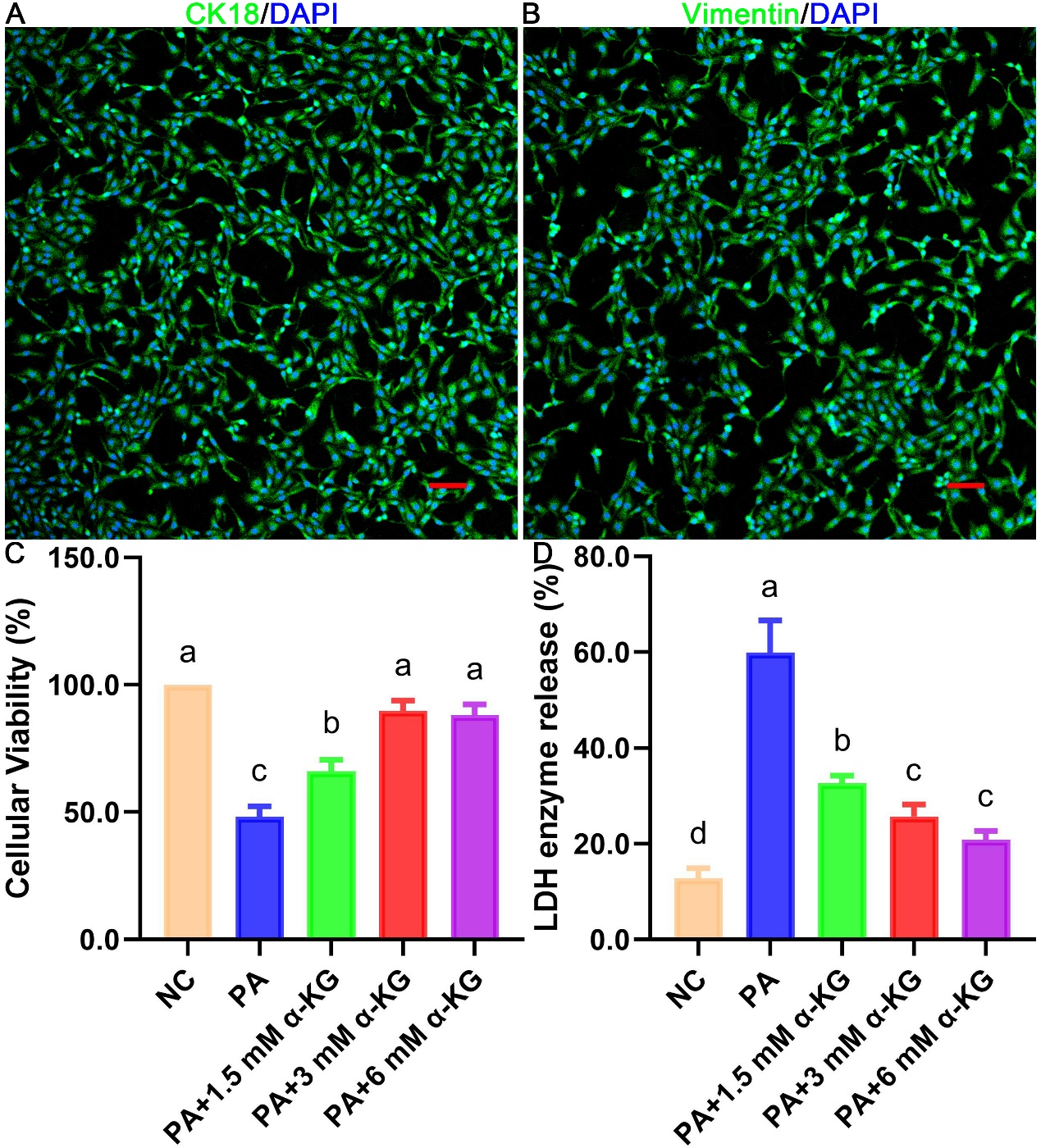


**Supplementation Figure 2. Protective Effect of α-KG on PA-Induced Cell Cycle Disruptions of SEECs**


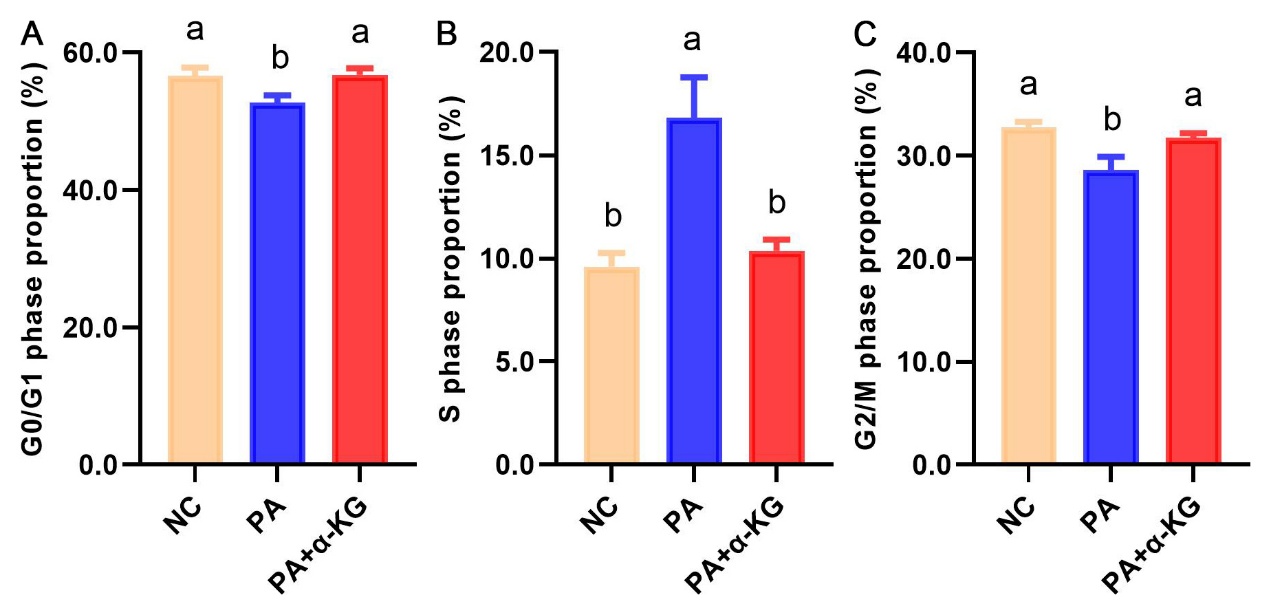


**Supplementation Figure 3. Pearson Correlation, Principal Component Analysis (PCA), and Gene Expression Profiling of Transcriptomic Data**


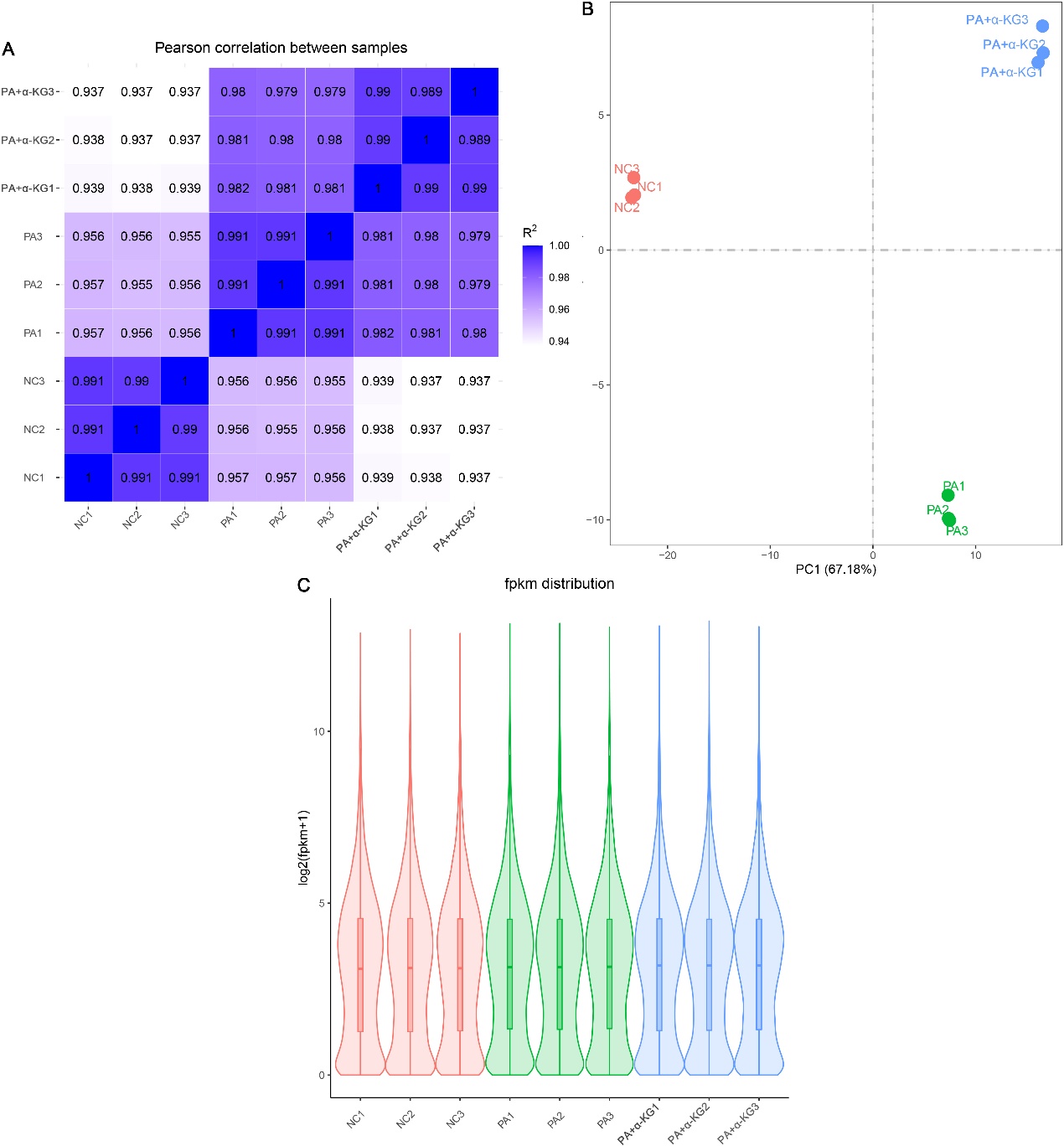


**Supplementary Figure 4. PCA and Partial Least Squares Discriminant Analysis (PLS-DA) of Metabolomic Data**


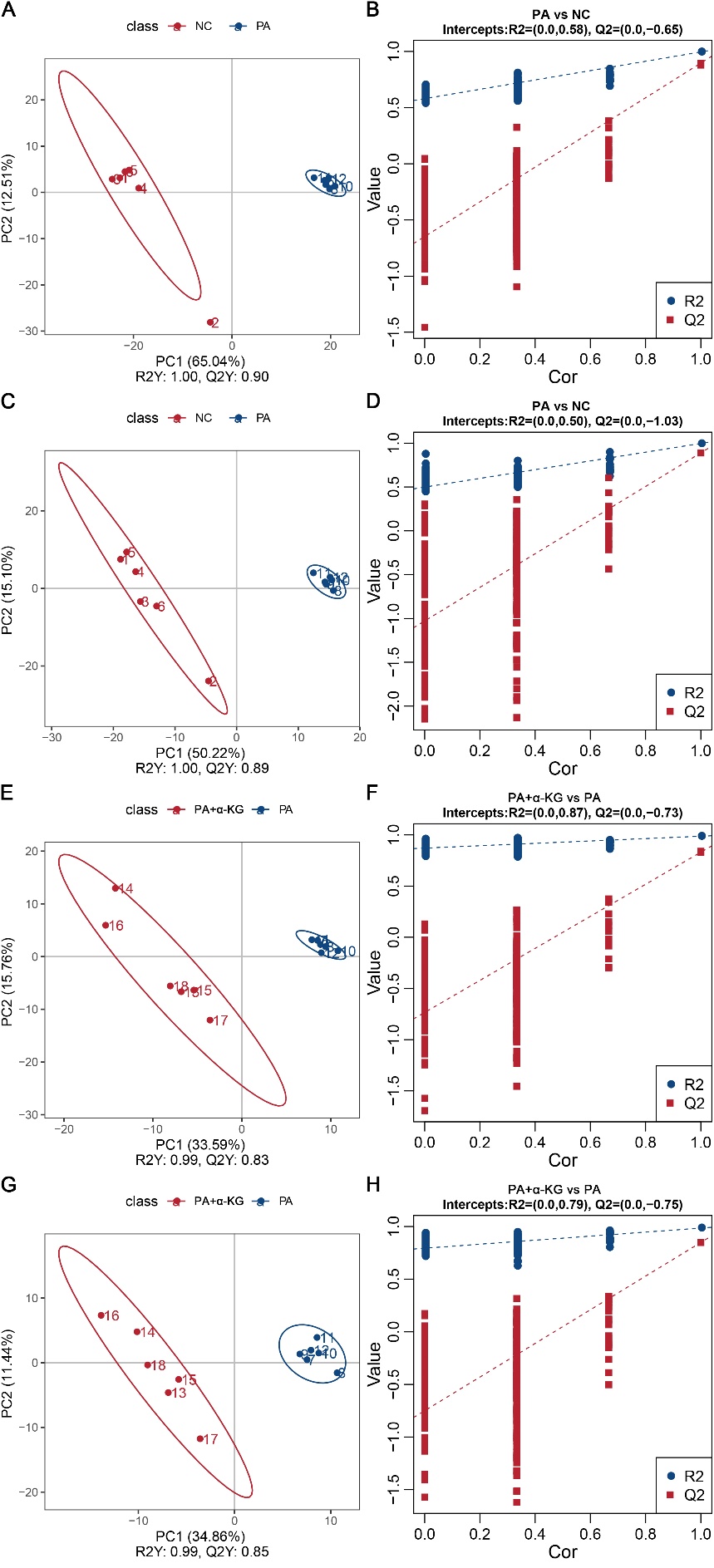


**Supplementation Table 1. Antibodies utilized in this study**

| **Antibody** | **Catalog number** | **Source** | **Application and dilution** | **Company name** |
| --- | --- | --- | --- | --- |
| CK18 | bs-1339R | Rabbit | IF staining, 1:500 | Bioss |
| VIMENTIN | bs-0756R | Rabbit | IF staining, 1:500 | Bioss |
| BAX | GB15690 | Rabbit | Western blot, 1:1000 | Servicebio |
| γH2A.X | ab81299 | Rabbit | IF staining, 1:500 | Abcam |
| CASPASE 3 | GB11767C | Rabbit | Western blot, 1:1000 | Servicebio |
| H3K4me3 | PTM-613 | Rabbit | IF staining, 1:500; Western blot, 1:2000 | PTM BIO |
| H3K9me3 | PTM-616RM | Rabbit | IF staining, 1:1000; Western blot, 1:5000 | PTM BIO |
| H3K27me3 | PTM-647RM | Rabbit | IF staining, 1:500; Western blot, 1:2000 | PTM BIO |
| H3K36me3 | BS-3768R | Rabbit | IF staining, 1:500; Western blot, 1:5000 | Bioss |
| SOD2 | 24127-1-AP | Rabbit | Western blot, 1:10000 | Proteintech |
| GPX4 | bsm-61552R | Rabbit | Western blot, 1:2000 | Bioss |
| SLC7A11 | 26864-1-AP | Rabbit | Western blot, 1:1000 | Proteintech |
| ACSL4 | 22401-1-AP | Rabbit | Western blot, 1:1000 | Proteintech |
| ACTIN | GB15003 | Rabbit | Western blot, 1:3000 | Servicebio |
| Histone H3 | PTM-1002RM | Rabbit | Western blot, 1:1000 | PTM BIO |
| Goat Anti-Rabbit IgG H&L (HRP) | bs-0295G | Goat | Western blot, 1:2000 | Bioss |
| Donkey Anti-Rabbit IgG H&L (Alexa Fluor® 555) | ab150074 | Donkey | IF staining, 1:500 | Abcam |
| Goat Anti-Rabbit IgG H&L (Alexa Fluor® 488) | ab150077 | Goat | IF staining, 1:500 | Abcam |
